# Translation, cultural adaptation and psychometric evaluation of the age-friendly cities and communities questionnaire for Sweden

**DOI:** 10.1186/s12877-026-08690-2

**Published:** 2026-11-13

**Authors:** Nina Buer, Jeroen Dikken, Joost van Hoof, Helen Lindner

**Affiliations:** 1 School of Health Sciences, Faculty of Medicine and Health, Örebro University, Örebro, 701 82 Sweden; 2 Research Group of Urban Ageing, Faculty of Social Work & Education, The Hague University of Applied Sciences, Johanna Westerdijkplein 75, Den Haag, 2521 EN the Netherlands; 3 Department of Systems Research, Faculty of Spatial Management and Landscape Architecture, Wrocław University of Environmental and Life Sciences, ul. Grunwaldzka 55, Wrocław, 50-357 Poland

**Keywords:** Age-friendly, City, Community, Confirmatory factor analysis, Rasch analysis

## Abstract

**Background:**

The Age-Friendly Cities and Communities Questionnaire (AFCCQ) measures how older people perceive the age-friendliness of their city or community. With 23% of Swedish residents projected to be aged ≥ 65 years by 2040, a culturally adapted and psychometrically validated Swedish version (AFCCQ-SE) is needed to guide age-friendly initiatives nationally. This study aimed to translate the AFCCQ into Swedish and to conduct its cross-cultural adaptation and psychometric validation.

**Methods:**

In Phase I, the AFCCQ-EN was independently forward-translated into Swedish and back-translated into English. Ten older adults and ten academic experts then rated each item’s relevance on a 4-point scale. In Phase II, 874 community-dwelling Swedes aged ≥ 65 years (64.9% female; mean age 75.7 ± 5.8 years; 82.8% urban) from two regions completed the AFCCQ-SE. Confirmatory factor analysis (CFA) was used to assess the nine-domain structure against fit criteria and composite reliability (CR) was used to evaluate internal consistency. Rasch analysis examined various aspects of the AFCCQ at the item and rating scale level.

**Results:**

I-CVI values of all items ranged from 0.80 to 1.00 (mostly 0.90). CFA supported the proposed nine-domain structure (χ²[194] = 562.9; normed χ² = 2.90; CFI = 0.962; TLI = 0.951; SRMR = 0.0335; RMSEA = 0.047). CR values were acceptable across most domains (0.783–0.937), with two domains marginally below the threshold (0.658–0.699). Rasch analyses showed acceptable rating categories and satisfactory item fit. Urban–rural DIF was found for two transportation items (0.51 & 0.71 logits, Holm adjusted *p =* 0.000). PCA supported unidimensionality within each domain rather than for the questionnaire as a whole. Twenty-two item pairs showed local dependence (*r* = 0.16–0.84), 21 of them within a single domain. Aggregating the items into nine testlets largely resolved local dependence (largest residual correlation = 0.10), and the first residual contrast eigenvalue decreased from 3.32 to 1.52 (< 2.0), supporting unidimensionality of the testlet model.

**Conclusions:**

This AFCCQ-SE study, using both CFA and Rasch analysis, provided preliminary support for its psychometric properties in this Swedish sample. Further validation using samples from larger regions and longitudinal study designs is needed to verify these findings.

**Supplementary Information:**

The online version contains supplementary material available at https://doi.org/10.1186/s12877-026-08690-2.

## Background

Population aging and rapid urbanization are two of the defining social shifts of the 21st century, placing new demands on the design and function of cities and communities. Age-friendly cities and communities are ones that actively promote health, inclusion and cohesion across all ages and levels of functioning. Such environments enable people to stay active, connected and able to contribute socially, culturally and economically, while also putting in place mechanisms to support those at risk of social isolation [1].

The World Health Organization’s (WHO) Age-Friendly Cities and Communities’ framework can be used as the conceptual cornerstone for understanding age-friendly cities and communities [2]. This framework emphasizes the interaction between urban environments and the well-being of older adults and was built on the earlier foundations of the active aging agenda [3]. Age-friendly cities and communities offer policies, services and structures related to the physical and social environment that are designed to support and enable community-dwelling older people to age actively. The framework consists of eight domains that influence the lives of older people living in the community, namely housing, social participation, respect and social inclusion, civic participation and employment, communication and information, community support and health services, outdoor spaces and buildings and transportation [2]. The framework draws from environmental gerontology theories, which highlight the dynamic relationship between older individuals and their physical, social, and service environments to promote health, participation, and security [4].

In 2015, the WHO’s Age-Friendly Cities and Communities’ initiative introduced a set of core indicators [5] but these metrics did not reflect older adults’ real-world experiences, which was at the centre of the original Age-Friendly Urban Community Project that led to the publication of *Global Age-Friendly Cities; A Guide* in 2007 [2]. The absence of a rigorously validated quantitative instrument encompassing all eight WHO domains, along with the framework itself lacking formal validation [6, 7] posed many challenges to cities wishing to join the global network and to evaluate the progress of their efforts and actions. To address this, researchers created the Age Friendly Cities and Communities Questionnaire (AFCCQ) [8], a 23-item tool covering the eight WHO domains as defined in the Global Age-Friendly Cities Guide [2], plus an additional financial domain [9]. While initially developed in the Netherlands, it is crucial for the questionnaire to undergo a thorough cross-cultural validation process in the country where the AFCCQ will be used, given the diverse cultural and socio-economic contexts of cities worldwide.

Sweden is a country in Northern Europe, where around 20% of the Swedish population were 65 years or older in 2019 and this number is projected to rise to 23% by 2040 [10]. Most older adults (96%) live at home, of which about 40% live alone [11]. Swedish cities and communities focus on creating age-friendly environments that support accessible housing, mobility, and social inclusion, while leveraging innovations such as digital healthcare to enhance the daily lives of their ageing populations.

Although previous Swedish studies have assessed various aspects of age-friendly environments, such as accessible housing, transportation, and social participation [12–14], there was a lack of a psychometrically valid questionnaire like the AFCCQ that assesses multiple aspects of age-friendliness within a single questionnaire. A culturally adapted and psychometrically evaluated Swedish version of the AFCCQ will enable comparisons between cities and communities, facilitate the planning and evaluation of age-friendly initiatives in Sweden. This study therefore aimed to translate the English AFCCQ into Swedish and carry out a cross-cultural adaptation and psychometric validation of the Swedish AFCCQ. It was hypothesised that the AFCCQ-SE would retain the original nine-domain structure and provide preliminary support for its psychometric properties in a Swedish sample.

## Methods

### Study design

The methodological design consisted of two parts: Part I – translation and cross-cultural adaptation of the AFCCQ-English (AFCCQ-EN) into Swedish and Part II – psychometric validation of the AFCCQ-Swedish (AFCCQ-SE).

### The AFCCQ

The English version of the AFCCQ (AFCCQ-EN) was developed by Dikken et al. in 2020 and it comprises 23 items categorized into nine domains [8]. The domains encompass “*Housing* (1, 2), “*Social participation* (3–6),” “*Respect and social inclusion* (7, 8),” “*Civic participation and employment* (9, 10),” “*Communication and information* (11, 12),” “*Community support and health services* (13–17),” “*Outdoor spaces and buildings* (18, 19),” “*Transportatio*n (20, 21),” and “*Financial situation* (22, 23).” Respondents rate each item on a 5-point Likert-type scale, ranging from totally disagree (-2) to totally agree (+ 2). Higher AFCCQ scores indicate higher perceived age-friendliness.

### Part I: Translation and cross-cultural adaptation of the AFCCQ-EN

Part I was carried out according to the steps from Sousa [15]. Two translators (a gerontology and planning expert and a linguist) independently translated the AFCCQ-EN into Swedish, a third translator then compared their versions with the original to produce a preliminary AFCCQ-SE. Once the Swedish researchers (first and last author) approved the version, two blinded translators back-translated it into English, and the original developers (second and third author) reviewed and approved it.

In line with COSMIN recommendations for content validity assessment [16], a panel of ten older Swedes rated each item’s relevance and comprehensibility (1–4), and a panel of ten gerontology researchers rated each item on relevance and comprehensiveness (1–4). Item Content Validity Indices (I-CVIs) were calculated for both panels, with values above 0.78 indicating excellent content validity [17]. The S-CVI/Ave was calculated as the mean of the 23 I-CVI values, while the S-CVI/UA was calculated separately for each panel as the proportion of items rated as relevant by all members of that panel.

### Part II: Psychometric validation of the AFCCQ-SE

**Fig. 1:**
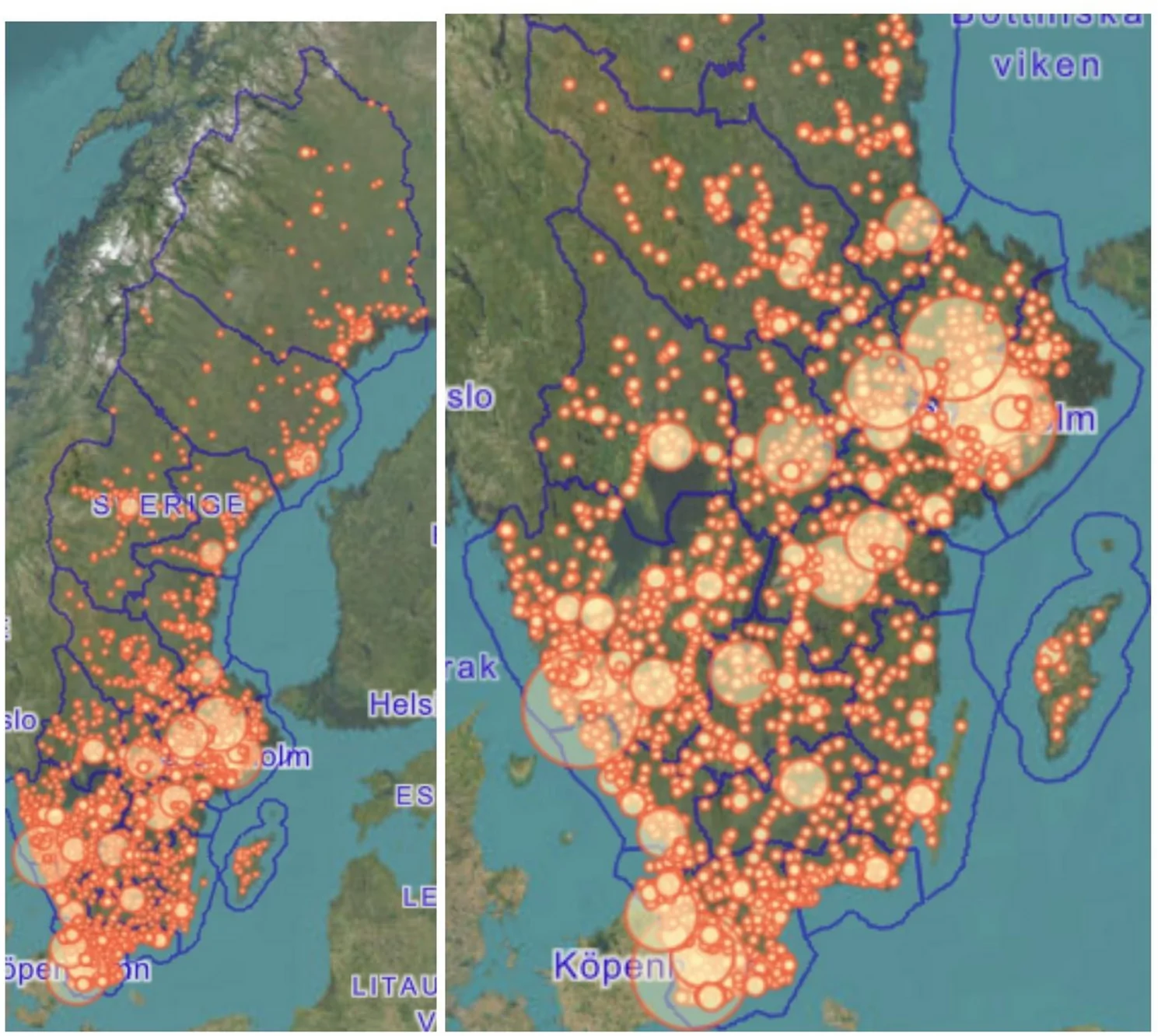
Distribution of urban areas (orange) in whole Sweden (left) and south Sweden (right) (source: SCB Tätorter avgränsningar och befolkning)

Sweden has an area of 450,000 km², which ranks it among the five largest countries in Europe. The total population of Sweden was 10,606,999 in year 2024 and 88% of them lived in urban areas (Fig. 1). Data collection was carried out in two lower central regions of Sweden: Region Örebro and Region Dalarna. Örebro and Dalarna cover 8,517 km² and 28,030 km² respectively. In 2024, the regions had populations of 308,375 and 286,546, while 68,324 (22.2%) and 74,684 (26.1%) were aged 65 or above. Among the inhabitants of age 65 or above in these two regions, around 52.9% were female.

An ethical application was submitted to the Swedish Ethical Review Authority on April 24, 2024, and the authority’s decision, received on May 28, 2024, confirmed that an ethical review was not required under Swedish law, as no personal data were collected (application number 2024-02840-01). One study inclusion criterion was being aged ≥ 65 years without a diagnosis that impaired their memory or decision-making capacity. Between June and December 2024, information about the project, a written consent form, and the AFCCQ-SE were sent to various organizations for people aged 65 and older, as well as to municipal meeting clubs for older adults in the two regions. These organizations then shared the materials with their members and attendees, who were invited to participate by completing the questionnaire. Three research assistants attended different organizational meetings and assisted the participants in completing the questionnaire. Informed consent to participate was obtained from all participants prior to completing the AFCCQ-SE. The procedures were carried out in compliance with the Declaration of Helsinki and ethical guidelines of the Swedish Ethical Review Authority. A total of 874 adults ≥ 65 years completed the AFCCQ-SE (Table 1).

**Table 1:** Demographic information of the participants

Demographics	Sweden (*n* = 874)
Sex	
Male n, %	306 (35.0%)
Female n, %	567 (64.9%)
*Missing value n*,* %*	1 (0.1%)
**Age**, Mean (SD)	75.69 (sd 5.83)
**Living in an urban area n**,** %**	724 (82.8%)
**Living in a rural area n**,** %**	150 (17.2%)

#### Confirmatory factor analysis

A confirmatory factor analysis (CFA) was performed on all the 874 participants to confirm whether the Swedish data fit the model proposed by Dikken [8]. This factor size is well above commonly recommended thresholds for CFA [18, 19]. Factors were allowed to co-vary by setting the variance to unity. To assess model fit, the following indices were used: the normed χ^2^ (≤ 5), the Comparative Fit Index (CFI) and Tucker-Lewis Index (TLI) (both ≥ 0.9), the Standardized Root-Mean Squared Residual (SRMR) (< 0.08), and the Root-Mean Square Error of Approximation (RMSEA) (< 0.08 for moderate fit). To assess internal consistency, composite reliability was used with values above 0.70 indicating appropriate reliability. After assessing model fit, initial age-friendliness scores were compared across sex, age groups and urban versus rural residence. Composite reliability was then tested.

#### Comparison between groups

Measurement invariance (MI) across sex (male/female) and residence (urban/rural) was tested to establish whether the AFCCQ-SE operated equivalently across these subgroups. A hierarchical sequence of increasingly constrained multi-group confirmatory factor models was estimated: configural invariance (equal factor structure), metric invariance (equal loadings), scalar invariance (equal loadings and intercepts), and strict invariance (equal loadings, intercepts, and residuals). Invariance was considered supported when changes in fit indices between successive models met the criteria proposed by Chen (2007) [20]: ΔCFI ≥ 0.010, ΔRMSEA ≤ 0.015, and ΔSRMR ≤ 0.030 (metric) or ≤ 0.010 (scalar and strict). The CFA was performed using IBM SPSS Amos version 29.0 [21], while descriptive statistics and group comparisons of age-friendliness scores across sex, age groups and urban versus rural residence were performed using IBM SPSS statistics version 29.0 [22].

#### Rasch analysis

A Rasch rating scale model analysis was then performed to examine the psychometric properties of the AFCCQ-SE at both the scale and item levels using Winsteps (5.3.4.0) [23]. The basic principle of the Rasch model is that a person’s probability of endorsing an item response depends on the difference between the person’s level on the underlying construct and the item’s difficulty. In the Rasch model, person and item raw scores are sufficient statistics for their respective parameters. Winsteps used joint maximum likelihood estimation to estimate person measures and item difficulties simultaneously. The resulting estimates were expressed on a common interval-level logit scale. Person measures and item difficulty measures are expressed in the same logit unit and could therefore be displayed together on a common scale, illustrating the distribution of participants’ perceived age-friendliness relative to the range of the AFCCQ items for measuring age-friendliness. The present study included 874 participants, exceeding commonly cited sample sizes for stable Rasch parameter estimation [24, 25]. This large sample increased the precision and robustness of the measures and enabled adequately sized subgroup analyses of differential item functioning. This was particularly valuable for evaluating the AFCCQ-SE items across different subgroups in the sample.

The Rasch model provided a range of metrics and criteria to examine various aspects of the AFCCQ.

##### Rating categories

The five rating categories were examined according to four criteria [26]: (i) there should be at least 10 responses in each rating category, (ii) the average AFCCQ person measure should be lower in a category representing low AFCCQ than in one representing high AFCCQ, (iii) the transition point between each two categories (threshold) should follow an increasing level of the underlying construct, and (iv) the category outfit mean square should be less than 2.0. The rating scale graphs generated by Winsteps were used to examine the ordering of thresholds and how the rating categories were positioned along the underlying construct.

*Item technical quality* was evaluated using infit and outfit mean-square (MnSq) statistics and their standardized Z-values (Zstd) [27]. These item fit statistics are based on the residuals (observed minus expected responses) and indicate how well each item fits the Rasch model. Values close to 1.0 indicate that the item fits the model expectation, whereas higher values (> 1.5) suggest underfit, indicating that the item may measure a different construct, be poorly worded, be overly broad, or have problematic response categories. Lower values (< 0.5) indicate overfit, suggesting that the item responses are overly predictable [28]. Consistent with previous recommendations, acceptable item fit was defined as MnSq values between 0.5 and 1.5 and Zstd values between − 2.0 and + 2.0 [25].

*Item separation*,* along with person separation*, helps to evaluate the effectiveness of an instrument in both assessing the differences between individuals and distinguishing between items. The item separation index gives an estimate (in standard error units) of the spread or “separation” of items along the measurement construct; the person separation index gives an estimate of the spread or separation of persons along the measurement construct. A separation index of at least 2.0 coupled with a reliability of 0.80 or higher is generally recommended [27]. Moreover, the item difficulty range and the person ability range should align well with each other (so-called *targeting*). A perfectly targeted instrument would have a difference of zero, whereas a difference of more than 1 logit indicates significant mistargeting [29].

*Differential item functioning* was assessed using Winsteps’ built-in DIF procedure, which computed DIF contrasts for two subgroups (sex; urban/rural residence). Winsteps anchored person measures at their estimates from the overall calibration and estimated item difficulties separately for each subgroup, thereby placing subgroup-specific estimates on a common measurement scale. DIF contrasts were tested using Welch’s t-tests. To control the error rate across the 23 item-level DIF tests for each subgroup, *p*-values were adjusted using the Holm procedure in SPSS syntax [22, 30].

*Unidimensionality* was used to investigate whether the items in the AFCCQ collectively measured “age-friendly cities and communities as a whole.” A principal components analysis (PCA) of residuals (the differences between the observed responses and the responses expected by the Rasch model) was used to determine whether all the items captured the same underlying construct [31]. According to Rasch criteria, unidimensionality is supported when at least 60% of the raw variance is explained by the measures and the first contrast’s unexplained variance is below 5% (or eigenvalue < 2.0, i.e. the strength of 2 items) [32].

*Local item dependence* was assessed using standardized residual correlations between item pairs. The mean correlation across all unique item pairs was calculated, and correlations exceeding the observed mean by more than 0.20 were considered indicative of local dependence [33]. Dependent items were aggregated into domain-level testlets, and the same criterion (mean + 0.20) was applied to the testlets.

## Results

### Part I​

#### AFCCQ-SE translation, adaptation, and initial validation

The forward translation was mostly performed correctly by the two translators with different areas of expertise, though several words required discussion. For example, the term neighbourhood (which appeared seven times in the AFCCQ-SE) was translated using two similar words. The term “närområde” was chosen because it emphasizes the physical area close to a person’s home rather than the social concept of a neighbourhood (grannskap). It was therefore easier for older adults to determine physical area rather than social concept of a neigbourhood. Additionally, the word “supply” in the item “*The supply of care and welfare in my city is enough for me*” did not align well with Swedish care and welfare and was therefore changed to “availability”. The final back translation closely resembled the original version and was approved by the original developer. The words that were translated differently and the final version of the AFCCQ-SE are available in Appendix 1 and 2 respectively.

Ten residents from the two regions (5 males, 5 females, aged 65 to 72 years), along with ten academic experts from universities in three different cities (5 males, 5 females, aged 55 to 70 years), assessed the relevance and readability of the AFCCQ-SE items. They specialized in geriatric nursing, community care, social care and urban planning. The I-CVI values ranged from 0.89 to 1.00 among the academic experts and from 0.80 to 1.00 among the older adults. The corresponding S-CVI/Ave values were 0.98 and 0.97, while the S-CVI/UA values were 0.78 and 0.70, respectively. One item, “*I sometimes get annoyed about negative remarks because of my age*,” has an I-CVI of 0.80 among the residents. No comments were made on this item. Three items had an I-CVI of 0.89. Two of them were items on” Housing” (item 1 and 2) and the experts were mainly concerned with the term “accessible.” For example, two written comments asked, “What is accessibility? For whom?” However, since these two “Housing” items were considered highly relevant by residents (I-CVI = 1.0) and no comments were provided regarding readability, they were kept unchanged. The I-CVI for item 14, “*When I am ill*,* I receive the care and help I need*,” was also 0.89. Two experts questioned, “How do I know if the care I receive is the care I need?” Nevertheless, item 14 received an I-CVI of 1.0 from residents, indicating high relevance. Thus, this item was also retained without changes.

### Part II​

#### The CFA results

The CFA model resulted in an acceptable fit (CFI = 0.962; TLI = 0.951; SRMR = 0.0335; RMSEA = 0.047) (Table 2). The composite reliability (CR) values for the constructs indicated a generally strong internal consistency across the dataset (Table 3). Most constructs met or exceeded the commonly accepted threshold of 0.7, including *housing* (0.783), *social participation* (0.859), *respect and social inclusion* (0.828), *communication and information* (0.827), *community support and health services* (0.874), *transportation* (0.920), and *financial situation* (0.937), highlighting their reliability and consistency in measuring the intended latent constructs.

However, *civic participation and employment* (0.699) and *outdoor spaces and buildings* (0.658) fell slightly below the 0.7 threshold. While this is generally considered a borderline result, it is still acceptable according to some references that recommend a minimum CR of 0.6 [34]. For outdoor spaces and buildings, the result suggests that the construct may benefit from further refinement. The lower composite reliability may be partially explained by the contribution of Item 19: *“The stores in my neighborhood are sufficiently accessible with a walker or wheelchair.”* This item contributes less to the construct, likely due to the inclusion of data from rural areas. The disparity between urban and rural contexts could lead to inconsistent responses, which may affect the overall reliability scores of the construct.

**Table 2:** Fit of data from Sweden with the original model as described by Dikken et al. [8]

Model	χ^2^*(DF)*	Normed χ^2^	Comparative Fit Index (CFI)	Tucker Lewis Index (TLI)	Root-Mean Squared Residual (SRMR)	Root-Mean Square Error of Approximation (RMSEA)**
	562.9 *(194)*	2.902	0.962	0.951	0.0335	0.047 *(0.042–0.051)*

*sig level ≤ 0.001, **95% *(90% CI)*

**Table 3:** Reliability per domain of the AFCCQ-SE

Domains	Housing	Social Participation	Respect and Social Inclusion	Civic Participation and Employment	Communication and Information	Community Support and Health Services	Outdoor Spaces and Buildings	Transportation	Financial Situation
Composite Reliability	0.783	0.859	0.828	**0.699**	0.827	0.874	**0.658**	0.920	0.937

Bold: Below recommended values 0.7

### Comparisons between groups

Prior to comparing domain scores across subgroups, measurement invariance of the AFCCQ-SE for sex (male/female) and residence (urban/rural) met the criteria for strict MI, with all ΔCFI ≥ − 0.005, ΔRMSEA ≤ 0.002, and ΔSRMR ≤ 0.003 (Table 4). Full (strict) measurement invariance was therefore supported, indicating that the AFCCQ-SE functioned equivalently across sex and residence groups and that observed mean differences across the nine domains could be meaningfully interpreted as substantive group differences rather than measurement artefacts.

**Table 4:** Measurement invariance of the AFCCQ-SE for sex and residence (*N* = 873)

Model	Χ^2^ (df)	CFI	TLI	RMSEA [90% CI]	SRMR	ΔCFI	ΔRMSEA	ΔSRMR
Sex (male vs. female)								
Configural	699.79 (388)	0.961	0.949	0.043 [0.038-0.048]	0.037	-	-	-
Metric	716.95 (402)	0.961	0.951	0.042 [0.038-0.047]	0.039	-0.000	-0.001	0.002
Scalar	741.32 (416)	0.959	0.951	0.042 [0.038-0.047]	0.039	-0.001	0.000	0.000
Strict	772.41 (439)	0.958	0.952	0.042 [0.037-0.046]	0.041	-0.001	-0.001	0.002
Residence (urban vs. rural)								
Configural	776.57 (388)	0.953	0.939	0.048 [0.043-0.053]	0.038	-	-	-
Metric	804.81 (402)	0.951	0.939	0.048 [0.043-0.053]	0.041	-0.002	0.000	0.002
Scalar	856.99 (416)	0.947	0.935	0.049 [0.045-0.054]	0.042	-0.005	0.001	0.002
Strict	907.69 (439)	0.943	0.935	0.050 [0.045-0.054]	0.044	-0.003	0.000	0.002

Note. All values are robust (Satorra–Bentler scaled) estimates from multi-group CFA. χ² = scaled chi-square; df = degrees of freedom; CFI = Comparative Fit Index; TLI = Tucker–Lewis Index; RMSEA = Root Mean Square Error of Approximation with 90% confidence interval; SRMR = Standardized Root Mean Square Residual. Δ values reflect change relative to the previous (less constrained) model. Invariance is supported when ΔCFI ≥ − 0.010, ΔRMSEA ≤ 0.015, and ΔSRMR ≤ 0.030 (metric) or ≤ 0.010 (scalar/strict) [20]. All criteria were met at every level, supporting strict measurement invariance across both sex and residence

**Fig. 2:**
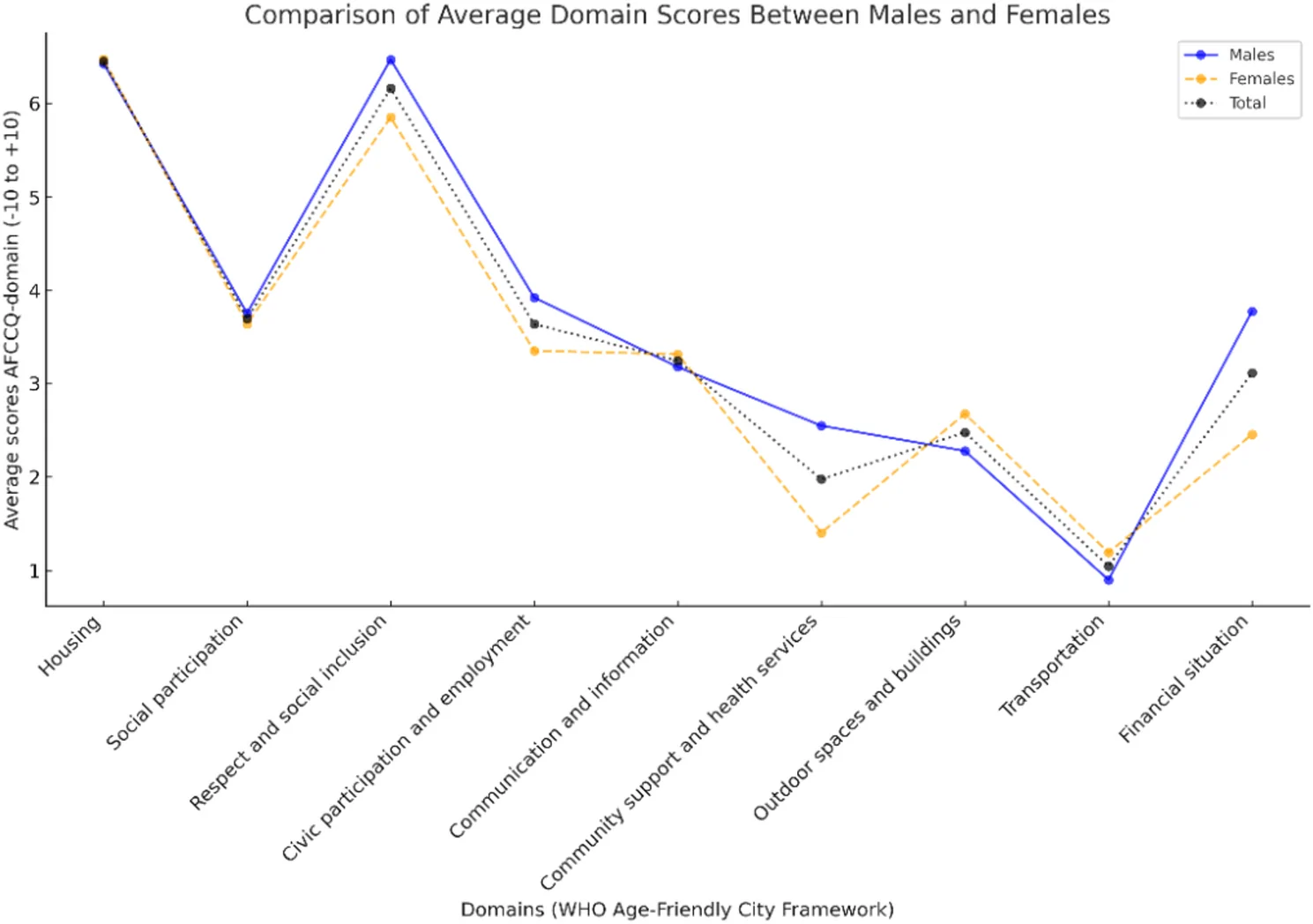
Comparison of average domain scores between males and females in who age-friendly domains

The comparison of average domain scores between males and females across the nine WHO age-friendly domains, as shown in Fig. 2, highlighted both similarities and significant differences. Males reported significantly higher scores in *Respect and Social Inclusion* (𝑝=0.048), *Community Support and Health Services* (𝑝<0.001), and *Financial Situation* (*p*<0.001) compared to females. No significant differences were observed between the sexes in the remaining domains. Notably, both groups demonstrated relatively high scores in *Housing* and *Respect and Social Inclusion*. In contrast, scores for *Community Support and Health Services*,* Outdoor Spaces and Buildings*,* Transport*, and *Financial Situation* were markedly lower across both sexes, with the domain of *Transportation* receiving the lowest average scores overall in Sweden.

**Fig. 3:**
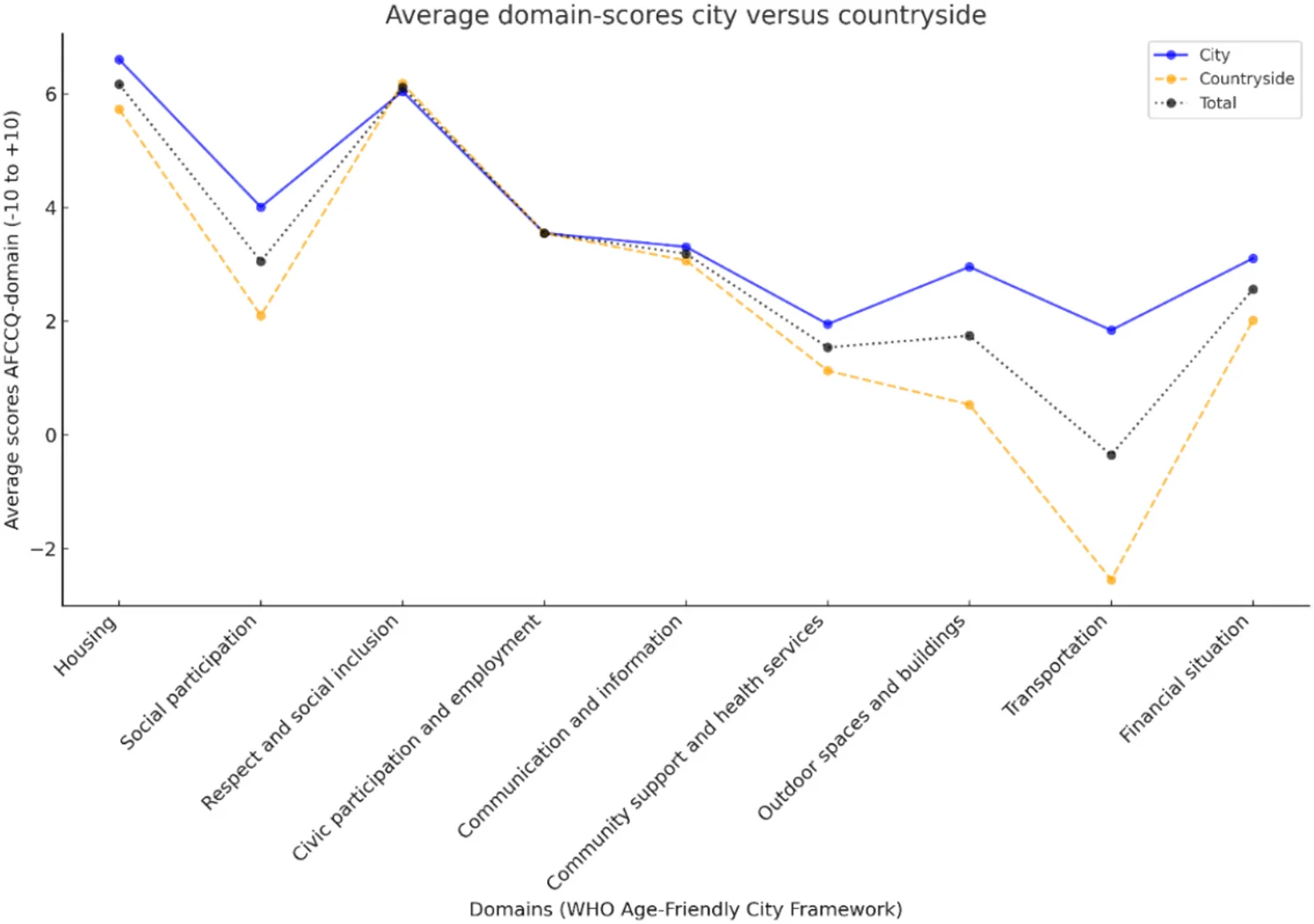
Comparison of average domain scores between people living in a city (urban) and people living at the countryside (rural) in WHO age-friendly domains

The comparison of average domain scores between urban and rural residents revealed significant differences in several domains, as shown in Fig. 3. Residents in the city reported significantly higher scores in *Social Participation* (*p* < 0.001), *Community Support* and *Health Services* (*p* = 0.037), *Outdoor Spaces and Buildings* (*p* < 0.001), *Transportation* (*p* < 0.001), and *Financial Situation* (*p* = 0.032) compared to their rural counterparts. Notably, the average score for *Transportation* in the rural areas was negative (mean score − 2.55), reflecting dissatisfaction with transportation infrastructure, whereas urban residents rated this domain positively (mean score 1.84). In contrast, no significant differences were observed for the remaining domains, including *Housing*,* Respect and Social Inclusion*,* Civic Participation and Employment*,* and Communication and Information*, which exhibited comparable scores between the two groups.

Overall, the data highlighted disparities between urban and rural environments, particularly in domains related to accessibility, infrastructure, and financial security, with city residents generally reporting more favorable conditions. However, the similarities in other domains suggested that certain aspects of age-friendliness, such as housing and social inclusion, were experienced similarly across both contexts.

### Rasch analysis results

#### AFCCQ logit scores in the sample

No significant difference (*p* = 0.070) was found between the mean AFCCQ person measure for men (0.86 logits) and for women (0.71 logits). The mean AFCCQ person measure for urban residents (0.82 logits) was significantly higher (*p* < 0.001) than the mean AFCCQ person measure for rural residents (0.46 logits).

#### The AFCCQ rating scale categories

**Fig. 4:**
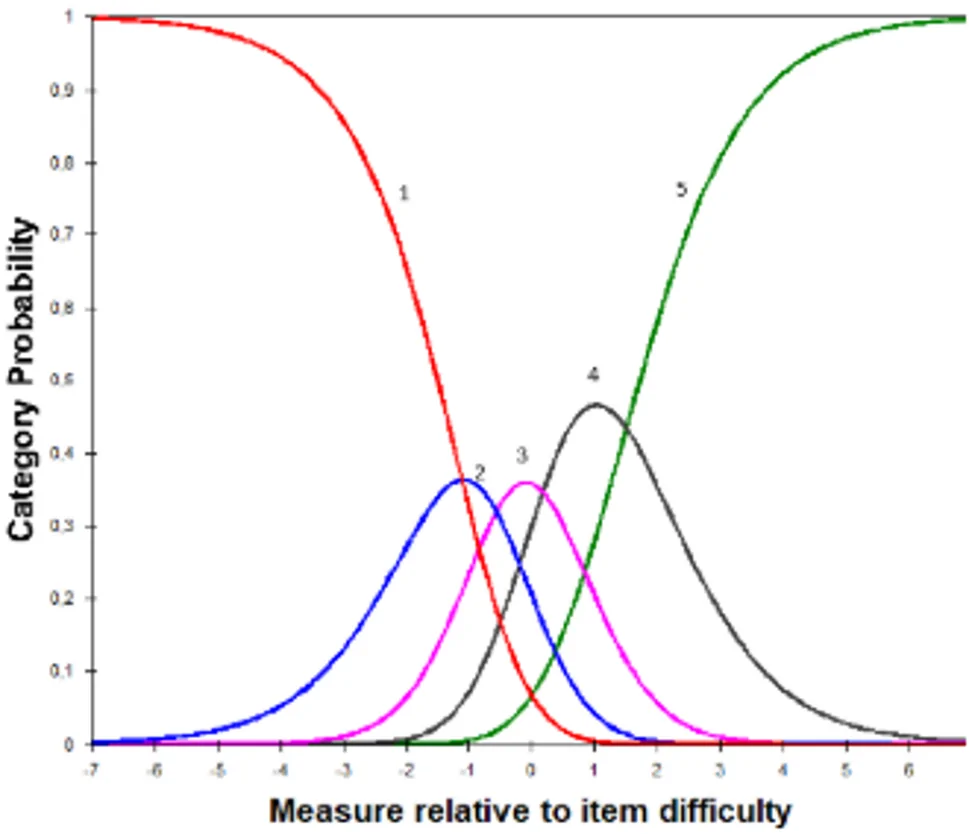
The category probability curves of the AFCCQ. The 1, 2, 3,4 and 5 category curves on the graph represent the five categories, from 1 = totally disagree to 5 = totally agree. They illustrate the range over which each of the five categories is most likely to be chosen. The x-axis is AFCCQ person measure minus AFCCQ item measure in logits

The five AFCCQ rating scale categories fulfilled the rating scale criteria (Table 5). That is, there were more than 10 responses in each rating category, the average person AFCCQ measure increased with the rating category, thresholds were ordered, and the category outfit mean square was below 2.0. Compared with the other three rating categories, Category 2 – “disagree” was selected in 11%, and Category 1 – “totally disagree” was selected in 4% of responses. The lower use of category 2 by the participants is visualized in the category probability curves (Fig. 4). The probability of Category 2 being selected (the blue curve) was lower than the probability of Category 4 “agree” being selected (the black curve).

**Table 5:** Summary statistics for the five AFCCQ rating scale categories

Category	Frequency of use (%)	Average AFCCQ person measure (logits)	Threshold measure	Category infit mean square	Category outfit mean square
1 - totally disagree	879 (4)	-0.30	NONE	1.05	1.24
2 - disagree	2266 (11)	0.08	-1.11	1.13	1.27
3 – neutral	4760 (24)	0.37	-0.56	1.00	1.05
4 – agree	7168 (36)	0.72	0.17	1.04	0.94
5 – totally agree	5029 (25)	1.57	1.50	0.87	0.91

#### Item fit statistics

Both Infit MnSq values and Outfit MnSq values of the AFCCQ items are within the recommended criteria (Table 6). This indicated that all items fitted the Rasch model as expected, functioning predictably and contributing to the measurement of the latent construct of “age-friendliness”.

**Table 6:** Fit statistics for AFCCQ items

Item*	Raw score**	Item measure (in logits)	Infit MnSq***	Infit Zstd****	Outfit MnSq***	Outfit Zstd****
Housing						
1. My house is accessible to me	3827	-1.11	1.47	7.32	1.36	5.40
2.My house is accessible to the people who come to visit me	3674	-0.76	1.44	7.27	1.43	6.80
Social Participation						
3.There are enough opportunities to meet people in my neighbourhood	3505	-0.45	0.89	-2.31	0.93	-1.39
4.Activities and events are organized in places that are accessible to me	3354	-0.20	0.89	-2.36	0.88	-2.36
5.The information about activities and events is enough for me and also suitable for me	3164	0.08	0.87	-2.81	0.91	-2.01
6. I find the range of events and activities sufficiently varied	3039	0.26	0.89	-2.53	0.96	-0.91
Respect and Social Inclusion						
7. I sometimes get annoyed or negative remarks because of my age	3795	-1.03	1.34	5.61	1.46	6.80
8. I sometimes face discrimination because of my age	3570	-0.56	1.34	5.97	1.35	5.81
Civic Participation and Employment						
9. I have enough opportunities to interact with younger generations	3327	-0.15	0.98	-0.49	1.03	0.68
10. I feel like a valued member of society	3158	0.09	0.69	-7.46	0.72	-6.39
Communication and Information						
11. Printed and digital information from the municipality and other social institutions is easy to read in terms of font and size	3146	0.11	0.85	-3.34	0.94	-1.33
12. Printed and digital information from the municipality and other social institutions is written in understandable language	3240	-0.02	0.81	-4.37	0.89	-2.34
Community Support and Health Services						
13. The supply of care and welfare in my city is enough for me	2904	0.43	0.97	-0.68	0.97	-0.68
14. When I am ill, I receive the care and help I need	3038	0.26	0.82	-4.14	0.83	-3.84
15. If necessary, I can easily reach care and welfare services by telephone and in person	2659	0.74	0.99	-0.18	1.01	0.27
16. I have enough information about care and welfare services in my neighbourhood	3002	0.31	0.71	-7.29	0.72	-6.65
17. Care and welfare workers in my neighbourhood are sufficiently respectful	3086	0.19	0.67	-8.09	0.74	-6.12
Outdoor Spaces and Buildings						
18. My neighbourhood is sufficiently accessible for a wheeled walker or wheelchair	3021	0.28	0.91	-1.97	0.95	-1.10
19. The shops in my neighbourhood are sufficiently accessible with a wheeled walker or wheelchair	3111	0.16	0.89	-2.41	0.99	-0.12
Transportation						
20. I can easily get on the bus or tram in my neighbourhood	2869	0.48	1.37	7.60	1.42	8.19
21.The bus and tram stops in my neighbourhood are easy to reach and use	2755	0.62	1.35	7.31	1.40	7.99
Financial Situation						
22. My income is sufficient to cover my basic needs without any problems	3176	0.07	1.22	4.38	1.30	5.62
23.I live well on my income	3088	0.19	1.16	3.32	1.22	4.43

Items*: the items are presented in the same order as in the AFCCQ

Total raw score**: sum score of each item

MnSq***= Mean Square standardized residuals, Zstd****= standardized Z-values

With all the AFCCQ items, the logit measures range from − 1.11 logits (*Item 1: My house is accessible to me*) to 0.74 logits (*Item 15: If necessary*,* I can easily reach care and welfare services by telephone and in person*), which is the most difficult item to endorse among the Swedish sample.

#### Separation indices

The person separation index was 2.51 (with reliability 0.86), indicating that the AFCCQ items worked well to separate the participants. The item separation index was 11.38 (with reliability 0.99), indicating that the AFCCQ items were capable of differentiating between different levels of perceived age-friendliness among the sample of 874 older adults.

**Fig. 5:**
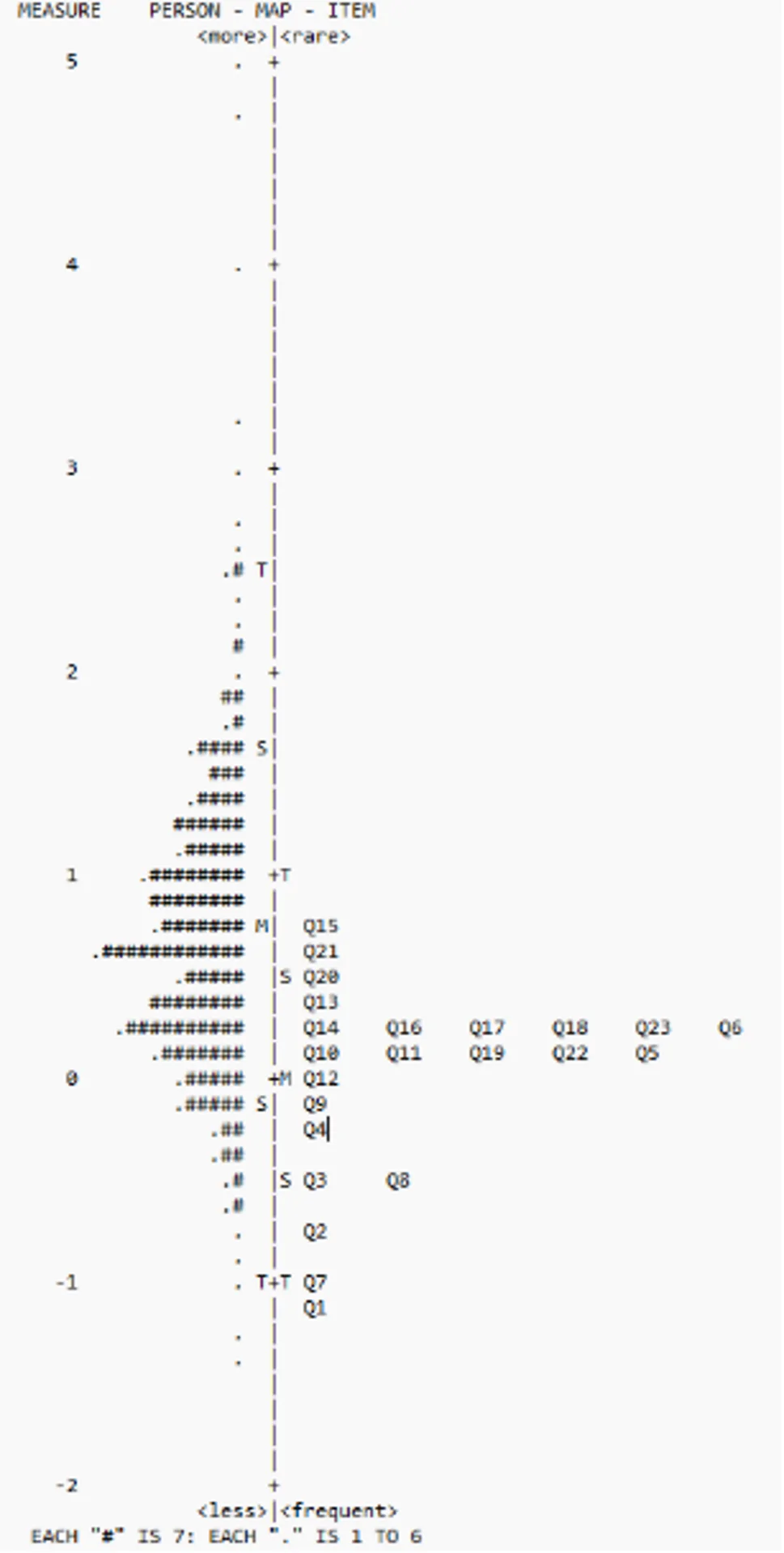
Person-item map: the AFCCQ items (Q1 to Q23) and the participants #

The person-item map (Fig. 5) visualized the spread of AFCCQ items in relation to the spread of the sample of 874 older adults along the same logit continuum. The mean item measure was centred at 0.00 logits by default, whereas the mean person measure was 0.76 logits. This person–item mean difference indicated fair targeting of the AFCCQ items to the sample.

#### Differential item functioning of the AFCCQ items

Table 7 presents the DIF contrasts and *p-*values for sex and place of residence. No item showed meaningful DIF between male and female participants, as all DIF contrasts were below 0.50 logits. After Holm correction, Items 3, 9, 17, 18, 20, and 21 showed significant urban–rural DIF. However, only the two Transportation items (Items 20 and 21) exceeded the 0.50 logit criterion (DIF contrasts = 0.71 and 0.51, respectively), indicating meaningful DIF. This implies that both items were easier for urban residents to endorse than for rural residents.

**Table 7:** Differential item functioning analysis between subgroups

Item*	Male vs. Female			Urban versus Rural		
	DIF contrast	*p*-value	Holm-adjusted *p*	DIF contrast	*p*-value	Holm-adjusted *p*
Housing						
1. My house is accessible to me	0.08	0.4536	1.0000	-0.03	0.8368	1.0000
2.My house is accessible to the people who come to visit me	0.13	0.1626	1.0000	0.00	1.000	1.0000
Social Participation						
3.There are enough opportunities to meet people in my neighbourhood	0.08	0.3430	1.0000	-0.43	0.0000	0.0000
4.Activities and events are organized in places that are accessible to me	0.10	0.2155	1.0000	-0.21	0.0364	0.4004
5.The information about activities and events is enough for me and also suitable for me	0.03	0.7341	1.0000	0.07	0.4756	1.0000
6. I find the range of events and activities sufficiently varied	0.07	0.3392	1.0000	-0.12	0.2052	1.0000
Respect and Social Inclusion						
7. I sometimes get annoyed or negative remarks because of my age	-0.12	0.2330	1.0000	0.38	0.0038	0.0608
8. I sometimes face discrimination because of my age	-0.14	0.1168	1.0000	0.34	0.0035	0.0595
Civic Participation and Employment						
9. I have enough opportunities to interact with younger generations	-0.07	0.3977	1.0000	0.34	0.0011	0.0209
10. I feel like a valued member of society	0.00	1.000	1.0000	0.29	0.0041	0.0615
Communication and Information						
11. Printed and digital information from the municipality and other social institutions is easy to read in terms of font and size	0.13	0.0888	1.0000	0.22	0.0254	​0.305
12. Printed and digital information from the municipality and other social institutions is written in understandable language	0.15	0.0715	1.0000	0.29	0.0041	​0.062
Community Support and Health Services						
13. The supply of care and welfare in my city is enough for me	-0.14	0.0733	1.0000	-0.05	0.6224	1.0000
14. When I am ill, I receive the care and help I need	-0.22	0.0057	0.1083	0.06	0.5537	1.0000
15. If necessary, I can easily reach care and welfare services by telephone and in person	-0.08	0.2989	1.0000	0.23	0.0145	0.1885
16. I have enough information about care and welfare services in my neighbourhood	-0.11	0.1646	1.0000	0.15	0.1034	1.0000
17. Care and welfare workers in my neighbourhood are sufficiently respectful	-0.24	0.0026	0.0520	0.31	0.0016	0.0288
Outdoor Spaces and Buildings						
18. My neighbourhood is sufficiently accessible for a wheeled walker or wheelchair	0.24	0.0015	0.032	-0.47	0.0000	0.0000
19. The shops in my neighbourhood are sufficiently accessible with a wheeled walker or wheelchair	0.16	0.0366	0.622	0.02	0.8076	1.0000
Transportation						
20. I can easily get on the bus or tram in my neighbourhood	0.25	0.0011	0.0253	**-0.71**	**0.0000**	**0.0000**
21.The bus and tram stops in my neighbourhood are easy to reach and use	0.12	0.1213	1.0000	**-0.51**	**0.0000**	**0.0000**
Financial Situation						
22. My income is sufficient to cover my basic needs without any problems	-0.18	0.0251	0.4518	0.00	1.0000	1.0000
23.I live well on my income	-0.26	0.0011	0.0253	0.08	0.3811	1.0000

Bold indicates meaningful DIF, defined as an absolute DIF contrast ≥0.50 logits and Holm-adjusted p < 0.05

#### Unidimensionality of the AFCCQ

The PCA of all 23 AFCCQ items indicated that the AFCCQ had more than one dimension. The raw variance explained by the measures (which should be above 60%) was 35.4% and the unexplained variance in the first contrast (which should be below 5%) is 9.3%. This supports that the AFCCQ itself is designed as a multidimensional scale.

**Fig. 6:**
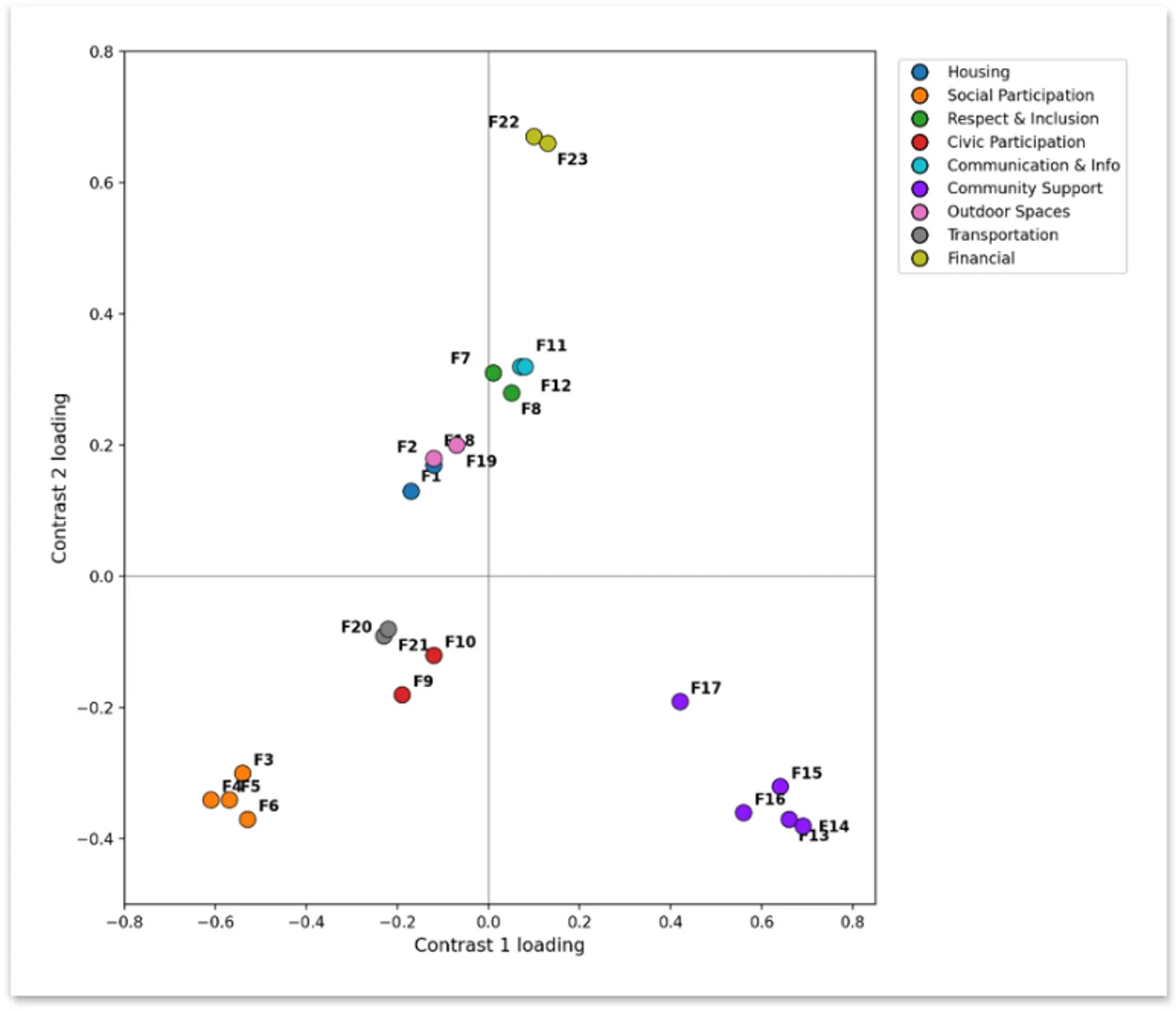
Item loadings on PCA Contrast 1 and 2 using 23-item AFCCQ-SE

Figure 6 plots the loadings on the first two contrasts. Some items clustered according to their intended domains, particularly Social Participation (F3–F6), Community Support and Health Services (F13–F17), and Financial Situation (F22–F23). Other domains showed overlapping patterns. For example, Housing and Outdoor Spaces and Buildings clustered together, possibly reflecting physical accessibility, while Civic Participation and Employment and Transportation showed similar loading patterns.

However, when each of the domains was analyzed separately, every domain fulfilled the criteria with raw variance explained by the measures 60.6% to 85.9% (Table 8). The unexplained variance in the first contrast of the domain “*social participation”* and “*community support and health services”* was higher than the recommended value 5%. However, the eigenvalues of both domains were 1.71 and 1.80 (which was below 2). This suggested that each domain fulfilled the criteria to be unidimensional.

**Table 8:** Principal component analysis of the AFCCQ domains

Domain	Raw variance explained by the measures	Unexplained variance in first contrast
Housing	61.9%	0.1%
Social participation	62.9%	**15.9%**
Respect and inclusion	72.5%	0.2%
Civic participation and employment	66%	0.0%
Communication and information	74.3%	0.0%
Community support and health services	60.9%	**14.2%**
Outdoor	60.6%	0.0%
Transportation	74.8%	0.0%
Financial situation	85.9%	0.0%

#### Local item dependence

The mean standardized residual correlation across all 253 unique item pairs was − 0.044, yielding a local-dependence threshold of 0.156. Twenty-two item pairs exceeded this threshold (*r* = 0.16–0.84; Table 9). Of these, 21 occurred within a single AFCCQ domain; only one pair (Q3–Q9, *r* = 0.16) spanned two domains: Social Participation and Civic Participation and Employment. Although assigned to different domains, both items concerned opportunities for social interaction: Q3 focused on meeting people, whereas Q9 focused on interacting with younger generations. Because all nine predefined domains contained at least one locally dependent pair and local dependence occurred almost entirely within domains, all 23 items were aggregated into nine domain-level testlets, one for each AFCCQ domain.

**Table 9:** Item pairs with standardized residual correlations exceeding the local-dependence threshold

AFCCQ Domain	Item pair	Standardized residual correlation (*r*)
Financial Situation	Q22–Q23	0.84
Transportation	Q20–Q21	0.76
Community Support and Health Services	Q13–Q14	0.64
Communication and Information	Q11–Q12	0.64
Respect and Social Inclusion	Q7–Q8	0.63
Social Participation	Q5–Q6	0.57
Housing	Q1–Q2	0.52
Social Participation	Q4–Q5	0.48
Community Support and Health Services	Q13–Q15	0.42
Community Support and Health Services	Q14–Q15	0.42
Social Participation	Q4–Q6	0.41
Community Support and Health Services	Q15–Q16	0.40
Social Participation	Q3–Q4	0.39
Outdoor Spaces and Buildings	Q18–Q19	0.35
Community Support and Health Services	Q14–Q16	0.34
Civic Participation and Employment	Q9–Q10	0.34
Community Support and Health Services	Q13–Q16	0.32
Community Support and Health Services	Q16–Q17	0.29
Social Participation	Q3–Q5	0.26
Social Participation	Q3–Q6	0.25
Community Support and Health Services	Q14–Q17	0.23
Social Participation and Civic Participation and Employment	Q3–Q9	0.16

In the testlet analysis, the mean standardized residual correlation was − 0.113, yielding a threshold of 0.087. Only the correlation between the Social Participation and Civic Participation and Employment testlets marginally exceeded this threshold (*r* = 0.10); all other residual correlations were ≤ 0.06. The raw variance explained by the measures increased from 35.4% to 47.0%, although it remained below the 60% criterion. The first residual contrast eigenvalue decreased from 3.32 to 1.52, meeting the < 2.0 criterion for essential unidimensionality. Person separation reliability decreased (real: 0.86 to 0.73; model: 0.89 to 0.80), whereas item/testlet reliability remained 0.99 in both analyses (Table 10).

**Table 10:** Comparison of key Rasch fit and dimensionality statistics for the 23-item and the 9-testlets

	23 item	9-testlet
Largest residual correlation	0.84	0.10
Raw variance explained by measures	35.4%	47.0%
1st contrast eigenvalue (unexplained variance)	3.32 (9.3%)	1.52 (9.0%)
Essential unidimensionality (Rasch/common variance)	52.2%	73.6%
Person separation reliability (real)	0.86	0.73
Person separation reliability (model)	0.89	0.80
Item/testlet reliability	0.99	0.99
Mean Infit/Outfit MNSQ (person)	1.04 /1.06	1.06 / 1.05
Mean Infit/Outfit MNSQ (item/testlet)	1.02 /1.06	1.01 / 1.05

## Discussion

This is the first study to psychometrically evaluate the Swedish, culturally adapted version of the AFCCQ using both classical test theory and item response theory through Rasch analysis. Content validity was generally adequate (CVI). CFA supported the original 9-domain structure, with fit indices within recommended thresholds. Rasch analysis showed acceptable rating scale functioning, item fit, and person/item separation. PCA indicated residual multidimensionality, and two transportation items showed urban–rural DIF. Aggregating items into nine testlets largely resolved local dependence. These findings support the hypothesis that the AFCCQ-SE would retain the original nine-domain structure and show preliminary support for its psychometric properties in a Swedish sample.

The content validity assessment of the AFCCQ-SE demonstrated that nearly all items were rated as highly relevant by both older adults and expert reviewers, yielding Item-CVI values predominantly in the 0.90–1.00 range. Two housing items (“*My house is accessible to me*” and “*My house is accessible to the people who come to visit me*”) received lower relevance scores from the experts due to the word “accessible”. Academic gerontology experts in Sweden consider accessibility not merely as the removal of physical barriers but also from whose perspective it is experienced [12, 35]. For older adults who live daily in their own homes, however, they have only their own perspective on the accessibility of their houses. Interestingly, these two housing items received the highest (Item 1) and third highest (Item 2) raw scores among all AFCCQ items (Table 6). One possible explanation is that Swedish older adults with health decline or illness generally live in homes that have already been adapted for accessibility, such as ramps, modified kitchens and bathrooms [36].

The CFA results supported the original 9-domain structure (the eight WHO age-friendly domains plus “Financial situation”), with goodness-of-fit indices well within recommended thresholds. This suggests that the hypothesized domain organization of the 23 AFCCQ items was confirmed in Sweden, mirroring the findings of the original AFCCQ [8]. When the AFCCQ was investigated as a whole, Rasch-based PCA of residuals supported multidimensionality, consistent with the instrument’s intended multidomain design; domain-level analyses confirmed that each domain was essentially unidimensional. As a residual-based diagnostic rather than a structure-confirming analysis, the item-loading plot (Fig. 6) was not expected to reproduce the nine-domain solution already established by CFA. Some domains formed tight, expected clusters (Community Support and Health Services; Social Participation), while items from conceptually related domains, such as physical accessibility (Housing; Outdoor Spaces and Buildings) and opportunities for engagement (Social Participation; Civic Participation and Employment; Transportation) loaded together. This suggests a plausible secondary pattern in the residual structure, consistent with content overlap between domains, rather than evidence against the nine-domain model, which remains supported by the CFA and domain-level PCA results.

At the item level, all items showed acceptable fit to Rasch model expectations, and the instrument demonstrated acceptable person and item separation. These results imply that the AFCCQ-SE can distinguish between older adults’ level of perceived age-friendliness and that the Swedish sample was sufficient to calibrate item difficulties with acceptable precision for the AFCCQ-SE. The five-category rating scale also functioned as intended, with thresholds advancing monotonically and no evidence of category collapse or disordering, indicating that respondents used the full response scale coherently. The person–item mean difference of 0.76 logits indicated fair, though not optimal, targeting. The AFCCQ-SE items were somewhat easier to endorse than the sample’s average level of perceived age-friendliness, suggesting lower precision among older adults reporting the highest levels of age-friendliness. Local dependence was observed among 22 item pairs, all but one within a single AFCCQ domain, and was largely resolved when items were aggregated into testlets. Such dependence may arise because items within the same AFCCQ domain assess closely related aspects and are therefore answered similarly. Although this is understandable conceptually, local dependence can inflate reliability and overestimate measurement precision. The one remaining marginal dependence, between the Social Participation and the Civic Participation and Employment testlets, is conceptually plausible, as both domains concern older adults’ social engagement, such as meeting people and interacting with younger generations.

Since the first validation of the original AFCCQ in the Netherlands [8], the AFCCQ has been professionally translated, culturally adapted, and psychometrically re-evaluated in multiple languages of different countries [37–49]. Comparing the Swedish results with the original Dutch validation and other country validations of the AFCCQ reveals certain similarities as well as some differences. The original AFCCQ was also reported to have excellent content validity (range 0.88–1.00) [8]. The Swedish CFA fit indices (CFI 0.962, TLI 0.951) align closely with those from Italy (CFI 0.968, TLI 0.959) and are slightly higher than those of the AFCCQ-Macedonia (CFI 0.877, TLI 0.840) [42, 46]. In the original Dutch validation, all nine domains were retained and showed satisfactory internal consistency (composite reliability ranging from 0.74 to 0.93), which is comparable to the reliability coefficients observed in this Swedish study. Slightly lower reliabilities were observed in the “*Civic Participation and employment*” (CR = 0.699) and “*Outdoor Spaces and Buildings*” (CR = 0.658) domains in the Swedish sample and in non-English speaking sample. The “*Civil Participation*” domain also had the lowest alpha among the nine domains in the Dutch sample. The borderline reliability observed in both samples could be due to the potential differences between the two items (item 9: *I have enough opportunities to interact with younger generations* and item 10: *I feel like a valued member of society*). Item 9 is about the interaction with younger adults whereas item 10 is about one’s perceived value in society.

A comparable structural explanation applies to the “Outdoor Spaces and Buildings” domain: its two items (Q18, Q19) capture perceived quality and accessibility of outdoor environments, but do not cover the full breadth of the WHO outdoor spaces domain, which also encompasses green spaces, walkability, safety, seating, and public toilets. Importantly, both of these lower-reliability domains are represented by only two items in the AFCCQ. Composite reliability, like Cronbach’s alpha, is mathematically dependent on the number of items in a scale: with only two indicators, even substantial inter-item correlations tend to yield relatively modest reliability estimates [50, 51]. This is therefore a structural feature of the AFCCQ design rather than a Sweden-specific finding. This structural feature also reflects the original design philosophy of the AFCCQ, which was explicitly conceived by Dikken et al. [8] as a compact ‘quick scan’ of age-friendliness. During its initial development, items that showed weaker factor loadings, or that were flagged during the face and content validity phases as culturally specific or ambiguous (for example items on green spaces), were removed to arrive at a parsimonious instrument. A consequence of this design choice is that several WHO domains are not exhaustively represented at the item level, and the full breadth of the underlying construct is therefore not fully captured (e.g. most domains capture accessibility). At the same time, this approach has a clear advantage for cross-cultural validation: the retained items are those that are most universally interpreted across contexts, which supports the international comparability of AFCCQ scores that has been demonstrated across multiple countries [28–40]. For applications requiring a more granular assessment of specific domains, for example, detailed evaluations of outdoor environments or civic engagement in a particular municipality, the original authors recommend to complement the AFCCQ with top-up questions or additional domain-specific scales, without compromising the core cross-cultural comparability of the instrument [8].

The Transportation domain (Q20, Q21) scored the lowest of all nine domains, and both items were rated lower by rural residents (as shown in DIF). This likely reflects genuine infrastructure differences rather than item bias: rural Swedish municipalities are often underserved by public transport, with essential services sometimes requiring over 30 min of travel time [52], and amenities are often sparsely distributed [53]. These constraints may be felt more acutely by older adults who rely on a walker, the most commonly used mobility aid among older Swedes [54, 55]. National “ageing in place” policies [56, 57] may therefore have uneven consequences depending on local transport infrastructure. This suggests that the DIF reflects a real difference in circumstances (item impact) rather than a flaw in the items themselves, with the practical implication that urban–rural comparisons within this domain should be interpreted with this context in mind.

### Methodological considerations

This study has several limitations. First, the use of convenience sampling may have introduced sampling bias, and the sample was drawn from only two regions, limiting the regional representativeness of the findings and their generalizability to the broader Swedish older adult population. Although the sample included more than 800 older adults, the participants in the sample lived in the middle part of Sweden. It is unknown whether the validation results would be similar if the sample would be living in large cities such as Stockholm or in the rural areas in northern Sweden. In terms of demographics, only age, sex and urban/rural areas were collected. This limits the comparisons between subgroups of respondents, such as education and ethnicity. Second, the self-administered nature of the questionnaire may have introduced response bias, as it relies on participants’ subjective interpretation and honest self-report without interviewer clarification. Third, the cross-sectional design did not allow evaluation of test-retest reliability and responsiveness to change over time, which should be addressed in future longitudinal studies. Furthermore, Sweden has advanced infrastructure and rapidly evolving technologies. The research team considered adding items on Swedish digitalisation to this version but decided to wait after this original version was psychometrically validated.

Two reasons motivated the use of both classical test theory and item response theory in validating the AFCCQ-SE. First, the original and subsequent international AFCCQ validations employed CFA, which enabled direct comparison of factor-analysis estimates across different AFCCQ studies. Second, Rasch analysis allows detailed investigation at the individual item level. Combining these methods is novel in age-friendly research; more importantly, this study shows that the AFCCQ remains robust regardless of the statistical approach. Such stability highlights the AFCCQ’s quality and supports its broader use in advancing age-friendly initiatives worldwide.

## Conclusions

Despite Sweden’s commitment to the WHO Global Network for Age-Friendly cities and communities, no instrument previously existed to assess age-friendliness in the Swedish context. This study presents the first psychometric evaluation of the AFCCQ-SE, demonstrating its potential use for measuring age-friendliness in Sweden.

## Supplementary information

Below is the link to the electronic supplementary material.

Supplementary Material 1

Supplementary Material 2

## Data Availability

The datasets used and/or analysed during the current study are available from the corresponding author upon reasonable request.
