# Supplementary material for: Translation, cultural adaptation and psychometric evaluation of the age-friendly cities and communities questionnaire for Sweden
Source: BMC Geriatr. 2026 Nov 13;26:1422. doi: 10.1186/s12877-026-08690-2 (PMC13761102; doi:10.1186/s12877-026-08690-2)
Supplement: Supplementary file 1 [file 12877_2026_8690_MOESM1_ESM.docx]

Appendix 1 - The wording differences in the original English version, the two Swedish translations, decision and reasons

| **Section/item** | **Original English** | **Swedish translation 1** | **Swedish translation 2** | **Final decision** | **Reason** |
| --- | --- | --- | --- | --- | --- |
| Q2 | the people who come to visit me | folk som kommer på besök | dem som besöker mig | Translation 2 | Simple to understand |
| Heading | Social participation | Social delaktighet | Deltagande i sociala sammanhang | Translation 1 | Less word, same meaning |
| Q3,16,17,18,19,20,21 | neighbourhood | grannskap | närområde | Translation 2 | Emphasizes the physical area close to a person’s home |
| Heading | Civic participation and employment | Samhällsdelaktighet och sysselsättning | Samhällsdeltagande och anställning | Translation 1 | A better choice considering the items under this heading |
| Q13 | The supply of care and welfare in my city | Tillgången till vård och stöd i min stad | Utbudet av vård och omsorg på min ort | Translation 1 | Align better with Swedish care and welfare |
| Q22 | sufficient to cover my basic needs | räcker till för mina basbehov | täcker mina grundläggande behov | Translation 2 | Closer to the original version |
| Q23 | live well | lever bra | lever gott | Translation 1 | More daily use |
