# Supplementary material for: Translation, cultural adaptation and psychometric evaluation of the age-friendly cities and communities questionnaire for Sweden
Source: BMC Geriatr. 2026 Nov 13;26:1422. doi: 10.1186/s12877-026-08690-2 (PMC13761102; doi:10.1186/s12877-026-08690-2)
Supplement: Supplementary file 2 [file 12877_2026_8690_MOESM2_ESM.docx]

|  | **Instämmer inte alls** | **Instämmer inte** | **Neutral** | **Instämmer** | **Instämmer helt** |
| --- | --- | --- | --- | --- | --- |
| **BOSTAD** |  |  |  |  |  |
| 1. Min bostad är lättillgänglig för mig |  |  |  |  |  |
| 2. Min bostad är lättillgänglig för dem som besöker mig |  |  |  |  |  |
| **SOCIAL DELAKTIGHET** |  |  |  |  |  |
| 3. Det finns tillräckligt med möjligheter att träffa människor i mitt närområde |  |  |  |  |  |
| 4. Aktiviteter och evenemang anordnas på platser som är lättillgängliga för mig |  |  |  |  |  |
| 5. Information om aktiviteter och evenemang är tillräcklig och passande för mig |  |  |  |  |  |
| 6.Jag tycker att utbudet av evenemang och aktiviteter är tillräckligt varierat |  |  |  |  |  |
| **RESPEKT OCH SOCIAL INKLUDERING** |  |  |  |  |  |
| 7. Jag får ibland negativa kommentarer på grund av min ålder |  |  |  |  |  |
| 8. Jag blir ibland diskriminerad på grund av min ålder |  |  |  |  |  |
| **SAMHÄLLSDELTAGANDE OCH SYSSELSÄTTNING** |  |  |  |  |  |
| 9. Jag har tillräckligt många möjligheter att träffa/umgås med yngre generationer |  |  |  |  |  |
| 10. Jag känner mig som en uppskattad samhällsmedlem |  |  |  |  |  |
| **KOMMUNIKATION OCH INFORMATION** |  |  |  |  |  |
| 11. Tryckt och digital information från kommunen och annan samhällsservice är lättläst vad gäller teckensnitt och storlek |  |  |  |  |  |
| 12. Tryckt och digital information från kommunen och annan samhällsservice är skriven på ett begripligt språk |  |  |  |  |  |
| **SAMHÄLLSSTÖD OCH HÄLSO- OCH SJUKVÅRD** |  |  |  |  |  |
| 13. Tillgången till vård och stöd i min stad är tillräcklig för mig |  |  |  |  |  |
| 14. När jag är sjuk får jag den vård och hjälp jag behöver |  |  |  |  |  |
| 15. Vid behov kan jag enkelt nå vård och stöd på telefon och personligen |  |  |  |  |  |
| 16. Jag har tillräckligt med information om vård och stöd i mitt närområde |  |  |  |  |  |
| 17. Vård- och omsorgspersonal i mitt närområde är tillräckligt respektfulla |  |  |  |  |  |
| **UTOMHUSUTRYMMEN OCH BYGGNADER** |  |  |  |  |  |
| 18. Mitt närområde är tillräckligt anpassat för en rollator eller rullstol |  |  |  |  |  |
| 19. Butikerna i mitt närområde är tillräckligt anpassade för en rollator eller rullstol |  |  |  |  |  |
| **TRANSPORT** |  |  |  |  |  |
| 20. Jag kan lätt komma på bussen eller spårvagnen i mitt närområde |  |  |  |  |  |
| 21. Buss- och spårvagnshållplatserna i mitt närområde är lätta att nå och använda |  |  |  |  |  |
| **EKONOMISK SITUATION** |  |  |  |  |  |
| 22. Min inkomst täcker mina grundläggande behov utan problem |  |  |  |  |  |
| 23. Jag lever bra på min inkomst |  |  |  |  |  |

© Nina Buer, med. dr, Helen Lindner, med. dr, Örebro Universitet 2025
